# ﻿How, not if, is the question mycologists should be asking about DNA-based typification

**DOI:** 10.3897/mycokeys.96.102669

**Published:** 2023-04-10

**Authors:** R. Henrik Nilsson, Martin Ryberg, Christian Wurzbacher, Leho Tedersoo, Sten Anslan, Sergei Põlme, Viacheslav Spirin, Vladimir Mikryukov, Sten Svantesson, Martin Hartmann, Charlotte Lennartsdotter, Pauline Belford, Maryia Khomich, Alice Retter, Natàlia Corcoll, Daniela Gómez Martinez, Tobias Jansson, Masoomeh Ghobad-Nejhad, Duong Vu, Marisol Sanchez-Garcia, Erik Kristiansson, Kessy Abarenkov

**Affiliations:** 1 Gothenburg Global Biodiversity Centre, Department of Biological and Environmental Sciences, University of Gothenburg, Box 461, 405 30 Göteborg, Sweden; 2 Department of Organismal Biology, Uppsala University, Norbyvägen 18D, 752 36 Uppsala, Sweden; 3 Chair of Urban Water Systems Engineering, Technical University of Munich, Am Coulombwall 3, 85748 Garching, Germany; 4 Mycology and Microbiology Center, University of Tartu, Liivi 2, 50409 Tartu, Estonia; 5 College of Science, King Saud University, 1145 Riyadh, Saudi Arabia; 6 Institute of Ecology and Earth Sciences, University of Tartu, Liivi 2, 50409 Tartu, Estonia; 7 Botany Unit (Mycology), Finnish Museum of Natural History, University of Helsinki, P.O. Box 7, FI-00014, Helsinki, Finland; 8 Department of Environmental Systems Science, ETH Zürich, Universitätstrasse 2, 8092 Zürich, Switzerland; 9 Interaction Design and Software Engineering, Chalmers University of Technology, Lindholmsplatsen 1, 417 56 Göteborg, Sweden; 10 Department of Clinical Science, University of Bergen, Box 7804, 5020 Bergen, Norway; 11 Department of Functional and Evolutionary Ecology, University of Vienna, Djerassiplatz 1, A-1030 Vienna, Austria; 12 Department of Biotechnology, Iranian Research Organization for Science and Technology, PO Box 3353-5111, Tehran 3353136846, Iran; 13 Westerdijk Fungal Biodiversity Institute, Uppsalalaan 8, 3584 CT Utrecht, Netherlands; 14 Department of Forest Mycology and Plant Pathology, Swedish University of Agricultural Sciences, Box 7026, 750 07 Uppsala, Sweden; 15 Department of Mathematical Sciences, Chalmers University of Technology, Göteborg, Sweden; 16 Natural History Museum, University of Tartu, Vanemuise 46, Tartu 51014, Estonia

**Keywords:** Dark taxa, ICN, nomenclature, species description, taxonomy, type principle

## Abstract

Fungal metabarcoding of substrates such as soil, wood, and water is uncovering an unprecedented number of fungal species that do not seem to produce tangible morphological structures and that defy our best attempts at cultivation, thus falling outside the scope of the International Code of Nomenclature for algae, fungi, and plants. The present study uses the new, ninth release of the species hypotheses of the UNITE database to show that species discovery through environmental sequencing vastly outpaces traditional, Sanger sequencing-based efforts in a strongly increasing trend over the last five years. Our findings challenge the present stance of some in the mycological community – that the current situation is satisfactory and that no change is needed to “the code” – and suggest that we should be discussing not whether to allow DNA-based descriptions (typifications) of species and by extension higher ranks of fungi, but what the precise requirements for such DNA-based typifications should be. We submit a tentative list of such criteria for further discussion. The present authors hope for a revitalized and deepened discussion on DNA-based typification, because to us it seems harmful and counter-productive to intentionally deny the overwhelming majority of extant fungi a formal standing under the International Code of Nomenclature for algae, fungi, and plants.

## ﻿Introduction

Dark matter is an astronomical concept that denotes mass of a hitherto unknown nature. That mass is detectable indirectly through the gravity it exerts – such as the bending of passing light – but its exact nature has so far defied scientific explanation. Mycology offers an analogy in the form of dark taxa, a concept that we define as taxa that do not seem to produce tangible morphological structures and that we cannot seem to cultivate in the lab (cf. Page 2016). As with dark matter, dark taxa are chiefly detected by means other than direct observation, notably through DNA sequencing (Grossart et al. 2016; Lücking et al. 2021). The field of mycology has become intimately entwined with the concept of dark taxa in the wake of environmental metabarcoding, where seemingly dark taxa often make up more than half of the taxa recovered (e.g., Retter et al. 2019). Dark taxa seem to permeate the fungal tree of life and are known from all major fungal lineages. Indeed, more than 20 class-level lineages of fungi seem to be constituted solely by dark taxa (Tedersoo et al. 2014, 2017, 2020a). Studying the fungal kingdom without its dark components is to study a paraphyletic group, something that contemporary phylogenetic thinking advises strongly against.

Most of the present authors have spent considerable time in the company of dark fungal taxa (DFT) as recovered through environmental metabarcoding and as manifested in the UNITE database for molecular identification of fungi (Nilsson et al. 2019; Abarenkov et al. 2022). The sheer magnitude of extant sequence data from DFT signals a need to take these taxa seriously. Yet it seems to the present authors that contemporary mycology often treats DFT as if they had a lesser – in fact, no – biological objectivity. The International Code of Nomenclature for algae, fungi, and plants (ICN; Turland et al. 2018) does not permit species descriptions typified from DNA sequences alone, and a recent effort to bring about change in this regard was overthrown by overwhelming majority (May et al. 2018). Similarly, DFT are routinely ignored in the context of, e.g., phylogenetic inference, ecology, and nature conservation (Ryberg and Nilsson 2018). Indeed, it is as if the DFT have no standing at all, scientific or otherwise. This goes very much against the experience of the present authors, who have used DFT to tease out branching orders, dominant but entirely overlooked taxa, and major ecological patterns that otherwise would have been lost on science (Nilsson et al. 2011, 2016; Khan et al. 2020; Tedersoo et al. 2022). Similarly, in an attempt to accord some taxonomic standing to the DFT, UNITE has assigned DOI-based digital identifiers to all DFT known from nuclear ribosomal internal transcribed spacer region (ITS) data to facilitate and promote unambiguous scientific communication across datasets and studies (Kõljalg et al. 2013). Although these DOI-based digital identifiers have been adopted by GenBank and the European Nucleotide Archive as LinkOuts to UNITE SH DOI pages – and were included in the Global Biodiversity Information Facility backbone classification already in 2018 (https://www.gbif.org/news/2LrgV5t3ZuGeU2WIymSEuk/adding-sequence-based-identifiers-to-backbone-taxonomy-reveals-dark-taxa-fungi) – these efforts have largely fallen short of sparking the debate they were hoping to.

In the present forum paper, we wish to visualize the relative contribution of DFT to molecular mycological species discovery over time. We do this through two molecular datasets, both of which reflect current knowledge but also biases in various ways. These datasets are: 1) all full-length fungal ITS sequences in the international nucleotide sequence database collaboration (INSDC; Arita et al. 2021) as of October 11, 2022 and 2) the five large metabarcoding datasets – chiefly of soil fungi (Ilves 2019; Tedersoo et al. 2020b, 2021; Lindahl et al. 2021; Runnel et al. 2022) – so far incorporated into the UNITE database. We find that the DFT overwhelmingly dominate the species discovery process, and it seems patently clear that extant fungal diversity presents us with patterns that cannot be accurately represented only by species defined by morphology or cultivation alone. It strikes us as unfortunate that what seems to be the absolute majority of fungi fall outside the scope of the ICN, and we hope that the present results will serve to add depth and dimension to the debate on how and when we should allow formal species descriptions based on DNA sequence data alone.

## ﻿Material and method

The full flow of operation behind the UNITE database is described elsewhere (Kõljalg et al. 2013, 2020; Nilsson et al. 2019). In brief, UNITE clusters the fungal ITS sequences of INSDC jointly with the UNITE-contributed DFTITS sequences into species hypotheses (SHs) at distance thresholds 0.5% through to 3.0% in steps of 0.5%. These operational taxonomic units can be thought of as entities roughly at the species level. The sequences and the SHs are available for web-based interaction as well as for download in various formats (https://unite.ut.ee/repository.php).

We downloaded all sequences included in the October 2022 version 9 release of the UNITE species hypothesis system. To allow us to contrast the species discovery from taxonomic and metabarcoding studies, we made the admittedly coarse assumption that all SHs that contained at least one sequence from the INSDC could be considered as taxonomy-derived SHs, that is, SHs with some sort of footing in traditional taxonomy. In contrast, all SHs containing only metabarcoding sequences were considered to be DFT. Based on the date of initial submission of each sequence (submission to INSDC and to UNITE, respectively, for INSDC and DFT sequences), we examined the accumulation of SHs over time. We plotted the accumulation of taxonomy-derived and DFT-only SHs against date of initial discovery in R v. 4.2.2 (R Core Team 2020). We similarly plotted the number of new fungal species descriptions per year (2002–2022) based on MycoBank (Robert et al. 2013), excluding recombinations, orthographic variants, invalid names, and illegitimate names.

While there is little hope of piecing together the ecological context of these sequences in an automated way, at least there is an opportunity to visualize the country of collection for many of the sequences in INSDC and UNITE. We thus sought to illustrate the geographical component of the SH accumulation curves by summarizing the country of collection of the taxonomy-derived and DFT sequences. In total, 63% of the taxonomy-derived, and 99.9% of the DFT, sequences were tagged with an explicit country of origin. The 20 most common countries of origin in each dataset were compiled using R.

## ﻿Results

**Figure 1.**
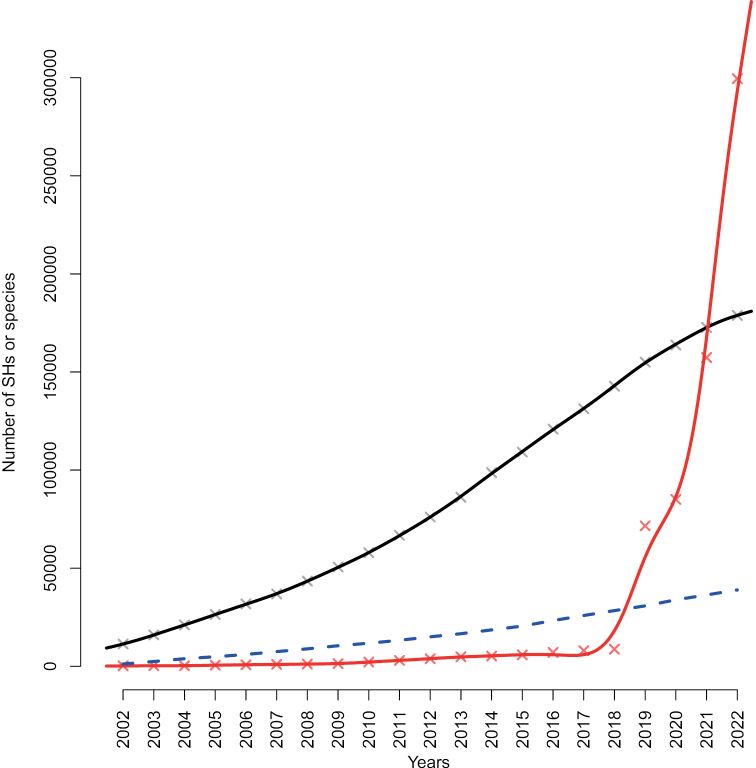
The accumulation of SHs at the 1.5% distance threshold over time in the Sanger (black; 88,665 studies of various sizes) and the DFT (red; 5 large studies) datasets. The Y axis depicts the number of SHs, and the X axis depicts year of sequence deposition. Solid trend lines were calculated using cubic smoothing splines. Also plotted (blue) is the cumulative number of newly described species for the period 2002–2022 (excluding recombinations, orthographic variants, invalid names, and illegitimate names). The numbers of species described in ca 2020–2022 may be slight underestimates due to widespread violation of the ICN recommendation F.5A to “inform the recognised repository of the complete bibliographic details upon publication of the name”. In reality, also the Sanger (INSDC) dataset is likely to hold some proportion of DFT. DFT sequences are notoriously difficult to tell apart in an automated way from sequences that are unidentified for other reasons (Abarenkov et al. 2022). The present study errs on the side of caution by treating all Sanger sequences as taxonomy-derived, meaning that Fig. 1 presumably under-estimates the proportion of DFT in the underlying data.

**Figure 2.**
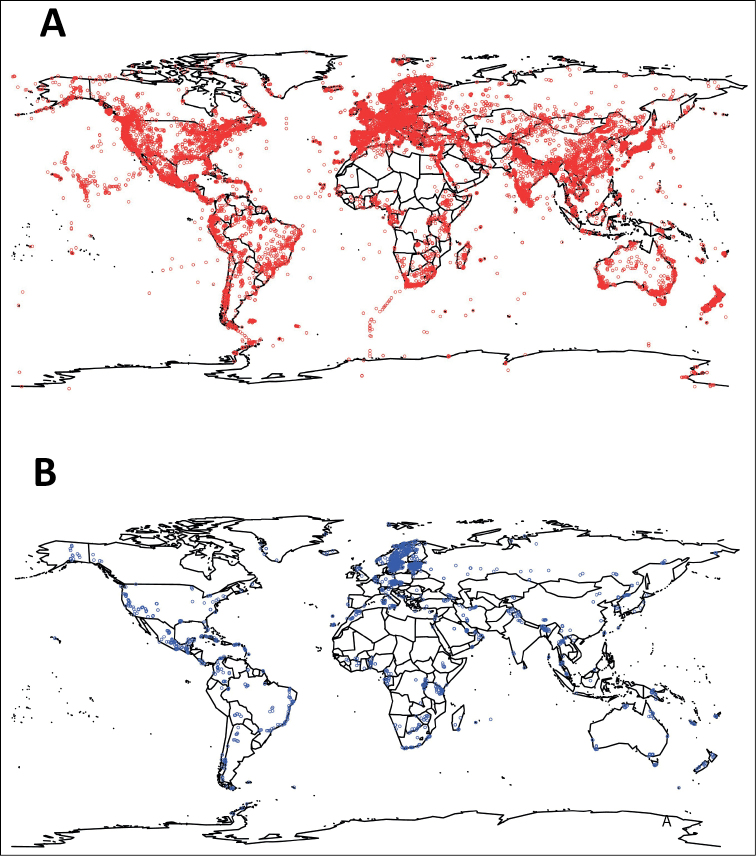
Maps showing the collection localities for the (**A**) Sanger sequences and (**B**) metabarcoding sequences that came with geo-coordinates (36,559 Sanger collection localities and 3,688 metabarcoding collection localities).

We retrieved a total of 1.26 M taxonomy- (Sanger sequencing-) derived sequences from INSDC and 7.1 M metabarcoding-derived DFT sequences from UNITE (https://unite.ut.ee/repository.php). The taxonomy-derived sequences were found to stem from a total of 88,665 distinct published and unpublished studies as defined by the combination of the INSDC fields AUTHORS, TITLE, and JOURNAL. The DFT sequences were found to stem from 5 studies. The SH accumulation curves at the dynamic 1.5% similarity threshold level are shown in Fig. 1, as is the number of new species of fungi described formally during the period. Table 1 shows the top 20 countries of origin for the taxonomy-derived and DFT sequences for which this data was available. Fig. 2 shows the collection localities for all Sanger and metabarcoding sequences with geo-coordinates.

**Table 1.** The 20 most common countries of collection for the Sanger and the DFT sequences. The DFT dataset is dominated by sequences from Estonia, from which most of the five metabarcoding studies were run. Estonia is not known as any particularly rich hotspot of biodiversity, perhaps suggesting that additional worldwide sampling would have produced even more dramatic increases in the number of DFT SHs.

INSDC country	INSDC seq.	DFT country	DFT seq.
Unknown	463524	Estonia	1788894
United States	133496	United States	350869
China	117292	Italy	287842
India	31788	Brazil	285473
Japan	29754	Czechia	260611
Brazil	27765	Russian Federation	228979
Canada	26038	Mexico	210643
Spain	22362	Norway	208422
Australia	22205	Colombia	204172
Germany	19971	Australia	177777
Italy	18078	Sweden	177318
Mexico	16326	Latvia	169168
France	14896	Lithuania	166553
Korea, Republic of	12434	Georgia	146440
Russian Federation	11668	Finland	127258
Iran, Islamic Republic of	11285	India	123706
Poland	10969	Argentina	116852
New Zealand	10956	China	100143
Thailand	10708	Papua New Guinea	96253
South Africa	10642	Tanzania, United Republic of	95203

## ﻿Discussion

The present study approximated fungal species accumulation over time as deduced from taxonomic and metabarcoding efforts. We found that the DFT account for the clear majority of the new species discovered in the last five years (although some limited proportion of both the Sanger-derived and the DFT sequences may possibly correspond to described, but so far unsequenced, species). We reached this conclusion based on a very limited number of metabarcoding studies – in fact, just five – of soil fungal communities and in almost complete absence of metabarcoding data from, e.g., water, air, wood, and plant material. One can only imagine that Fig. 1 would have shown an even more dramatic trend had a wider selection of metabarcoding datasets been available in UNITE. Fig. 2 paints a similar picture with respect to the geographical coverage. It shows that whereas the sampling effort of the five metabarcoding studies was wide, it pales in comparison to that of the combined Sanger-derived studies. It is reasonable to think that at least some of the unsampled geographical regions are rich in DFT and would have contributed to an even steeper trend in Fig. 1, had they been sampled.

When data are sparse, opinions may be maintained and cherished for longer than necessary. Our results show that data are no longer sparse; DFT, in view of their diversity and abundance, form a major, inextricable component of the fungal kingdom. They simply cannot be ignored. It is not scientifically defensible to exclude them from mycological efforts in phylogeny, ecology, or biogeography. We therefore argue that it does not make sense to deny them a formal standing under the ICN. We feel that it is time – in fact, long overdue – to resume and deepen the discussion initiated by, e.g., Hibbett et al. (2009, 2016), Hawksworth et al. (2016), and De Beer et al. (2016) and focus the debate on what the requirements should be for DFT to be formally considered under the ICN. Clearly, morphological structures or cultivability cannot be part of those requirements. We would like to reiterate the observation of Lücking et al. (2021) that a limited number of thought-through requirements would probably suffice. These should reflect the need for scientific reproducibility and should be stringent enough that only particularly well-vetted and documented DFT can be considered for DNA-based typification and formal description. At the same time, they should be realistic and reasonable enough that formal taxonomic description does become possible for such particularly well-vetted and documented DFT. We submit the following as tentative criteria:

- All DFT should be deduced and described based on the three nuclear ribosomal markers small-subunit (SSU/18S), the intercalary ITS region, and the large-subunit (LSU/28S). Of these, the ITS region is the most likely to reflect species-level distinctness in most groups of fungi. Parts of both the LSU and perhaps to a lesser extent SSU are likely to be at least somewhat informative of the species level in many groups of fungi, and unlike the ITS region they can typically be robustly aligned beyond the genus level in the pursuit of large-scale taxonomic affiliation. An initial recovery of an interesting ITS sequence can be extended to include SSU and LSU also from environmental samples using the design of specific primers as explored by Tedersoo et al. (2017) and Wurzbacher et al. (2019). In contrast, teasing out non-contiguous and/or non-ribosomal genes and genetic markers from complex, multi-species substrates such as soil or water in a way that ensures that the ribosomal, and the non-contiguous/non-ribosomal, markers come from the same individual is likely to remain problematic for the foreseeable future. We believe that allowing description of DFT based on any genetic marker would lead to irreconcilable species definitions and datasets. Having all DFT rooted in the ribosomal markers would provide a common ground from which mycology can proceed to solve taxonomic and nomenclatural issues and complications in a non-redundant way. The ribosomal markers will presumably not differentiate all DFT at the actual species level, and we imagine that additional genetic markers may have to be targeted (once technology allows) to reflect the species level properly. However, we don’t view that as a sustainable argument to allow DNA-based typification based on any genetic marker.
- A minimum length/coverage for the underlying sequence data, namely all of SSU, ITS, and LSU in their full lengths and in a contiguous stretch.
- Sufficiently high read quality.
- Stringent sequence quality control for, e.g., chimeras.
- At least two independent recoveries (each with some minimum read count in the case of metabarcoding) of the taxon across separate datasets from separate research teams from separate molecular laboratories.
- A thorough analysis of the public sequence databases for relevant additional sequences to maximize the penetration of available data and to minimize redundant descriptions.
- An underlying phylogenetic analysis based on a multiple sequence (SSU plus LSU) alignment. The new, contiguous SSU-ITS-LSU sequence(s) can then be incorporated into, e.g., a kingdom-wide SSU+LSU multiple sequence alignment obtained from merging the SSU+LSU alignments of James et al. (2006) and Tedersoo et al. (2018). This would allow reasonably robust phylogenetic positioning of the new sequence(s). A more fine-grained phylogenetic tree can be produced from ITS sequences only, or ITS+LSU, as alignability allows.
- Bundling of open, richly annotated raw sequence data/FASTQ/chromatograms and metadata on, e.g., the ecological and geographical specifics of the sampling sites.
- Publication in a peer-reviewed scientific journal with a formal impact factor or perhaps in any of a list of peer-reviewed journals revised annually by the Nomenclature Committee for Fungi or the International Commission on the Taxonomy of Fungi. The purpose would be to curtail mass description of DFT species through, e.g., self-publication or outlets not regularly considered by the mycological community.
- It furthermore seems reasonable to us to allow DNA sequences as types only in fungal groups that are predominantly or exclusively dark at, say, the order or supra-order level. We would be against DNA-based typifications in groups where morphological structures and/or cultivation may be within reach (e.g., *Agaricales* and *Hypocreales*). The order *Archaeorhizomycetales* may serve as an example of what a “predominantly” dark lineage could be. Soil sequencing has revealed an enormous species-level diversity within this order, yet cultures (and thus names) have been successfully worked out for only two species (Khan et al. 2020). The present authors are for DNA-based typifications in this order. While both *Agaricales* and *Hypocreales* can be expected to feature DFT, we are against DNA-based typification in these orders.
- At least one mycological taxonomist should be involved in the description of DFT (indeed, all fungi). There is no shortage of potential complications that, if overlooked, could lead to needless and haphazard introduction of new species and genera in DFT and beyond. For instance, it is well known that some extant genera offer examples of very divergent ITS (or other ribosomal) regions (e.g., *Basidiodendron*, *Oliveonia*, and *Cantharellus*; Feibelman et al. 1994; Alm Rosenblad et al. 2016, 2022). When considered in isolation and out of context – in, say, a molecular ecology dataset – such sequences could be incorrectly interpreted to warrant new species and genus descriptions. Needless to say, the present authors are against premature description of species and other taxonomic groups. While the concept of “a mycological taxonomist” may be amorphous, we submit the definition “someone who has [co-]authored at least one fungal species name as indexed in Index Fungorum/MycoBank/Fungal Names” for further discussion.

There is clearly room for refinement of the requirements mentioned here, and we are furthermore certain that the mycological community can come up with additional prerequisites to further increase stringency and reduce the risk for haphazard, more or less irreproducible or irresponsible use of DNA sequences as types (cf. Hibbett et al. 2016; Lücking et al. 2018; Zamora et al. 2018; Renner 2021). The present authors warmly welcome – indeed, invite – such a discussion. We understand that a special purpose committee for DNA-based typification under the auspices of the Nomenclature Committee for Fungi (http://www.ima-mycology.org/nomenclature/nomenclature-committee-fungi) will be put together and will hold an introductory meeting in April 2023. To that committee we would like to stress the urgency and the high stakes of the situation at hand.

It could be argued that a separate nomenclature code should be erected for the DFT, akin perhaps to the *Candidatus* concept in bacteria (Murray and Stackebrandt 1995; Pallen 2021) or to the extant DOI-based species identifiers of UNITE. We remain sceptical, however, and we argue for full-fledged integration of the DFT into the ICN. It seems likely to us that DFT as governed by a separate and more or less unofficial code or naming convention would simply remain relegated to some state of secondary – in practice, no – importance in the eyes of large parts of the mycological community and beyond. That is not the message delivered by Fig. 1, however, and not a state fit to reflect the crucial roles fungi are increasingly understood to play in the ecosystems of the world – by the scientific community and the general public alike. On the contrary, DFT seem to dominate the fungal kingdom. This puts the ICN in a position where it governs an ever-dwindling proportion of the extant fungi – maybe just some few percent. Such a position would seem untenable and, ultimately, vulnerable to usurpation. After all, the new and rebellious prokaryotic SeqCode (Hedlund et al. 2022) grew out of frustration at the inability of the International Code of Nomenclature of Prokaryotes (ICNP) to adapt enough to be able to reflect extant prokaryotic diversity properly. Naturally enough, the SeqCode makes no specific provisions for eukaryotes (or viruses, for that matter; Simmonds et al. 2017) at this stage, although we suppose that there is nothing – at least in principle – that would rule out such an expansion of target groups and inclusiveness over time. While the ultimate fate of SeqCode remains to be seen (Marinov et al. 2022), it does set an example of what the future may hold in store for ICN should DFT continue to be ignored. The present authors feel that it would be much better to modify the ICN than to consider more drastic actions. Specifically, we argue that formal scientific names for DFT are necessary for them to be taken seriously. Similarly, formal names will in practice be needed in biological conservation and in efforts exploring DFT for, e.g., medical and industrial use. These fungi deserve and need formal names, and it is our firm belief and opinion that this is achievable.
